# Perioperative intravenous lidocaine infusion improves postoperative analgesia after hysterectomy: a systematic review and meta-analysis of randomized controlled trials

**DOI:** 10.1097/JS9.0000000000001942

**Published:** 2024-07-08

**Authors:** Peng Tang, Qingxia Sun, Zhihao Li, Xiangyi Tong, Fengshou Chen

**Affiliations:** a The First Hospital of China Medical University Shenyang; b The First Clinical College, China Medical University Shenyang; c The Second Clinical College, China Medical University Shenyang; d Department of Anesthesiology, The First Hospital of China Medical University, Shenyang, Liaoning Province, People’s Republic of China

**Keywords:** hysterectomy, lidocaine, meta-analysis, opioid consumption, postoperative nausea and vomiting, visual analogue scale

## Abstract

**Background:**

The effectiveness of intravenous lidocaine in reducing acute pain after hysterectomy remains uncertain. The authors conducted a meta-analysis of randomized controlled trials (RCTs) to investigate the impact of intravenous lidocaine on posthysterectomy recovery.

**Methods:**

This study was completed based on the PRISMA guidelines and the Cochrane Handbook for Systematic Reviews of Interventions. A systematic search was conducted in PubMed/MEDLINE, the Cochrane Controlled Trials Register (CENTRAL), and Embase up to 27 July 2023. The authors identified RCTs involving hysterectomy patients comparing lidocaine to a placebo. Outcome measures included postoperative pain scores at rest and during movement, opioid consumption, postoperative nausea and vomiting (PONV), improvements in functional gastrointestinal recovery, and Quality of Recovery scores.

**Results:**

Nine RCTs were included in the meta-analysis, comprising 352 patients who received intravenous lidocaine and 354 controls. The analysis revealed that intravenous lidocaine significantly reduced postoperative pain scores at rest at 2, 6, 8, and 24 h following hysterectomy, as well as postoperative opioid consumption within 24 h and PONV rates. Furthermore, no observed benefit was noted in shortening the time to first flatus with intravenous lidocaine administration posthysterectomy.

**Conclusion:**

Intravenous lidocaine administration effectively reduces acute postoperative pain, opioid consumption, and PONV rates following hysterectomy. Lidocaine serves as an opioid-sparing agent, reducing the morphine equivalent dose while maintaining a similar degree of postoperative pain.

## Introduction

Highlights

- Intravenous lidocaine could reduce the severity of rest pain following hysterectomy.
- Intravenous lidocaine reduced opioid consumption following hysterectomy.
- Intravenous lidocaine decreased the nausea and vomiting following hysterectomy.

Following surgery, common immediate issues include postoperative pain, ileus, nausea and vomiting, postoperative cognitive dysfunction, wound infection, atelectasis, and hypercoagulation^1^. If acute postoperative pain remains untreated, it can impede postoperative recovery, prolong hospitalization, and elevate socio-economic burden^2^. Opioids serve as the primary agents for postoperative pain relief, administered intravenously (systemic analgesia) or through epidural catheters (epidural analgesia). However, opioid usage may heighten the risk of postoperative complications such as surgical site infection, urinary retention, pruritus, postoperative nausea and vomiting, sedation, and respiratory depression^3^. Hence, there is a necessity to explore alternative interventions to optimize perioperative care, thereby potentially enhancing the efficacy of existing analgesic approaches. Lidocaine, also known as lignocaine, was discovered in 1942 and introduced into clinical practice in the United States in 1949 and in Sweden in 1948^4^. Lidocaine is an aminoamide local anesthetic with antiarrhythmic effects. The inhibitory effect of intravenous lidocaine on postoperative pain was discovered and validated in 1951^5^. Lidocaine exhibits antihypersensitivity, anti-inflammatory, and analgesic properties, suggesting its potential as an alternative to opioids for alleviating postoperative pain^6^. Postoperative pain often presents as heightened sensitivity to pain, which may result from a combination of inflammatory and neuropathic factors^1^. The mechanism by which intravenous lidocaine alleviates postoperative pain may involve the inhibition of ectopic pulse discharge originating from the site of nerve injury and the interruption of dorsal root ganglion transmission^7^. The analgesic effect of lidocaine is mediated through the inhibition of spontaneous impulses from the proximal dorsal root ganglion and injured nerve fibers, involving the blockade of G protein-coupled receptors, N-methyl-D-aspartate (NMDA) receptors, and sodium channels. Its anti-inflammatory properties are attributed to the suppression of superoxide anion release and reduction in neutrophil adhesion^7^. A meta-analysis has shown that lidocaine infusion during abdominal surgeries, such as open colorectal surgery, major abdominal surgery, and laparoscopic cholecystectomy, can reduce the consumption of anesthetics and opioids intraoperatively and postoperatively^8^. Other clinically relevant outcomes affected by intravenous lidocaine administration include intestinal function recovery, postoperative cognitive impairment, coagulation, analgesia, and wound healing^9^. Considering the analgesic effect of intravenous lidocaine during the perioperative period, lidocaine infusion may improve perioperative outcomes and offer a safe adjunct to opioid-based analgesic strategies^1^. However, it is important to emphasize that lidocaine is used to supplement opioids rather than replacing them. The efficacy is determined by the extent to which the morphine equivalent dose is reduced while maintaining a similar level of postoperative pain. Previous meta-analyses have demonstrated the benefits of intravenous lidocaine in mitigating postoperative pain and reducing opioid consumption in patients undergoing various surgeries, including hepatectomy^10^, complex spine surgery^11^, and laparoscopic cholecystectomy^12^. However, due to limited research and sample size, the impact of intravenous lidocaine on the recovery and pain relief after hysterectomy has not been systematically evaluated^13–21^. Currently, hysterectomy procedures mainly include abdominal hysterectomy, laparoscopic hysterectomy, robotic total abdominal hysterectomy, and others. Therefore, we conducted a meta-analysis to evaluate the effectiveness of intravenous lidocaine in patients undergoing hysterectomy. The objective of this meta-analysis is to assess the effects of lidocaine infusion on hysterectomy, including postoperative pain, opioid consumption, gastrointestinal recovery, and opioid-related side effects such as postoperative nausea and vomiting (PONV).

## Method

### Study protocol

The meta-analysis was conducted and reported in accordance with the Preferred Reporting Items for Systematic Reviews and Meta-Analyses (PRISMA 2020) guidelines^22^ and Assessing the Methodological Quality of Systematic Reviews (AMSTAR)^23^. The protocol was registered on PROSPERO.

### Inclusive criteria

All studies meeting the predefined evidence-based Population, Intervention, Comparison, Outcomes, and Study Design (PICOS) criteria were included. These criteria comprised: (i) Patients: individuals undergoing hysterectomy, (ii) Intervention: administration of intravenous lidocaine, (iii) Comparator: use of normal saline via intravenous infusion, (iv) Outcomes: extraction of predetermined endpoints, including the primary outcomes of postoperative pain scores at rest and during movement and morphine consumption postsurgery, and secondary outcomes of the incidence of postoperative nausea and vomiting, Quality of Recovery scores (QoR-40), length of hospital stay, postoperative ileus, and functional gastrointestinal recovery (time to first bowel movement, time to first flatus, or time to defecation), and (v) Study design: randomized controlled trials (RCTs). Exclusion criteria included studies involving children, nonrandomized trials, studies not reported in English, nonoriginal studies (e.g. reviews and editorials), nonhuman studies, studies not related to hysterectomy, studies involving drugs other than lidocaine, and studies using lidocaine administration methods other than intravenous infusion, such as intraperitoneal administration.

### Literature search

The search for studies published up until 27 July 2023, was conducted in three electronic databases: PubMed/MEDLINE, Embase, and the Cochrane Controlled Trials Register (CENTRAL). Medical subject heading (MeSH) terms such as ‘lidocaine’ and ‘hysterectomy’ were used without language limitations. Two authors independently conducted the literature search, and any discrepancies were resolved through mutual agreement between them.

### Studies selection for meta-analysis

Duplicate studies were first excluded, and the remaining studies underwent two-stage screening^23^. In the initial stage, irrelevant research was excluded by reviewing the titles and abstracts. The second stage involved evaluating the full text of the remaining studies to select those for inclusion in the meta-analysis. The screening was independently conducted by two authors, and any discrepancies were resolved through discussion.

### Extraction of relevant data

Data extraction was independently performed by two authors. Two categories of data were extracted. The first category included baseline characteristics of the selected RCTs, such as author name, publication country, year, sample size, average age of participants, and detailed information on intravenous lidocaine infusion. The second category included outcome indicators of the study, such as pain scores during postoperative rest and movement, postoperative morphine consumption (mg), postoperative vomiting and nausea, QoR-40 scores, and functional gastrointestinal recovery (including time to first bowel movement and time to first flatus). Postoperative pain scores were measured at specified time intervals, and morphine consumption within 24 h postsurgery was evaluated. If the study results did not align with our specified time points, the closest available time point was selected for analysis^24^.

### Bias risk assessment

Two authors independently evaluated the methodological quality of each study included in the meta-analysis using the Cochrane bias risk assessment tool^25^. Standard risk of bias domains included personnel and outcome assessors, blinding of participants, allocation concealment, random sequence generation, incomplete outcome data, and selective reporting^1^. Each domain was categorized at the study level as having unclear, low, or high risk of bias.

### Data synthesis and meta-analysis

Data analysis was conducted using Review Manager (RevMan 5.3.4, The Cochrane Collaboration). The inverse variance method and the Mantel–Haenszel method were utilized to compare continuous data (postoperative pain scores, morphine consumption, QoR-40, time to first flatus, and bowel movement) and dichotomous data (rates of postoperative vomiting and nausea), respectively. Results for continuous data were presented as mean difference (MD) with 95% CI, while results for dichotomous data were presented as risk ratio (RR) with 95% CI.

Pain data reported on various scales, such as VAS 0–10 cm, VAS 0–100 mm, or NRS 0–10, were converted to VAS 0–10 cm for subsequent statistical analysis. Results were summarized as MD.

Opioid consumption data were converted to intravenous Morphine Equivalents (MEQ in mg) using a conversion method (http://www.whocc.no/atc_ddd_index).

Some continuous data were reported as means with median and interquartile range (IQR) or standard errors (SE). Following previous studies^26,27^, median with IQR and SE were transformed into mean with SD.

Heterogeneous and homogeneous results were analyzed using random effects and fixed effects models, respectively. Heterogeneity between studies was assessed using the *I* ^2^ test (>50%) and *χ* ^2^ test (*P*<0.05)^28^. Subgroup analysis and the one-out method were utilized to address heterogeneity between studies where possible.

## Result

### Results of literature search and summary of the included RCTs

**Figure 1:**
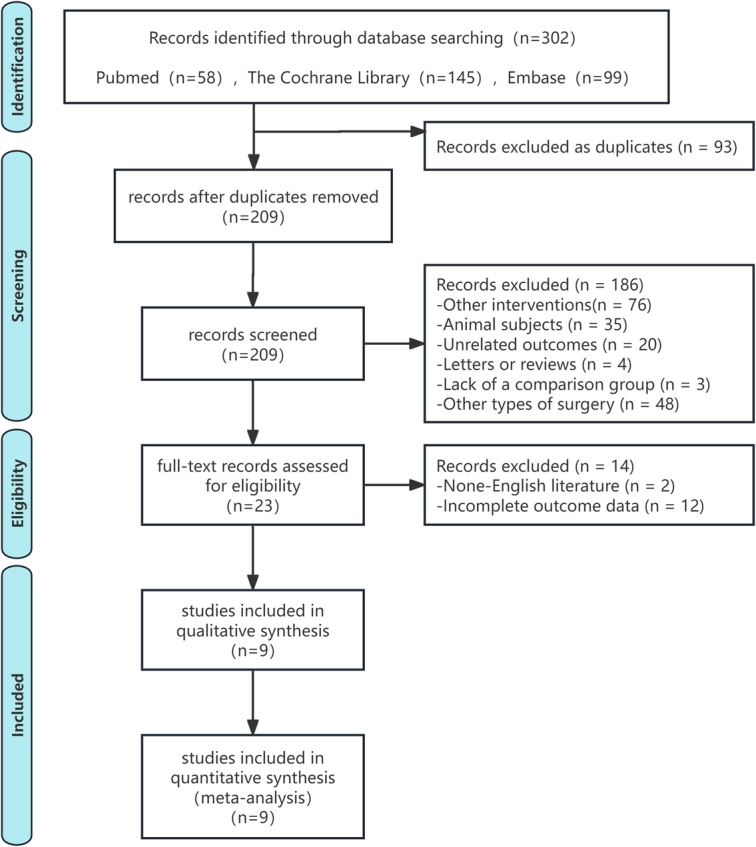
Flowchart of systematic search. As per PRISMA guidelines.

Nine RCTs were included in the meta-analysis^13–21^. The PRISMA flowchart depicting study selection is presented in Figure 1. A total of 706 patients were included in this meta-analysis, comprising 352 in the lidocaine group and 354 in the control group. All nine RCTs were published between 1987 and 2023 and conducted in single centers (Table 1), including locations in China^14,17,19,21^, Denmark^16^, India^15,20^, Canada^18^, and Israel^13^. Patients meeting the American Society of Anesthesiologists (ASA) Class I–II criteria were included in all nine studies. The surgical procedures involved abdominal hysterectomy in five trials^13,16,18–20^, laparoscopic hysterectomy in three trials^14,17,21^, and robotic abdominal hysterectomy in one trial^15^.

**Table 1:** Characteristics of studies included in this meta-analysis.

Reference	No. of patients		Type of surgery	Intervention	Control	Endpoints
	Lidocaine	Control				
Xu (2023)^21^	40	40	Laparoscopic hysterectomy	Lidocaine infusion(1.5 mg/kg for bolus over 10 min before induction of anesthesia, 1.5 mg/kg/h continuous infusion until 30 min before the end of surgery)	Placebo	Pain scores PONV QoR-40
Sivaji (2021)^15^	24	24	Robotic Hysterectomy	Lidocaine infusion (1.5 mg/kg loading over 15 min, 2 mg/kg/h continuous infusion till end of surgery)	Placebo	Pain scores Postop. opioid Time to first flatus
Xu (2021a)^14^	40	40	Laparoscopic hysterectomy	Lidocaine infusion(bolus infusion of 1.5 mg/kg over 10 min before induction of anesthesia, 1.5 mg/kg/h continuous infusion until 30 min before the end of the operation)	Placebo	Pain scores PONV Time to first flatus
Xu (2021b)^17^	60	60	Laparoscopic hysterectomy	Lidocaine infusion (bolus infusion of 1.5 mg/kg over 10 min prior to induction of anesthesia, 1.5 mg/kg/h continuous infusion until 30 min before the end of operation)	Placebo	Pain scores Postop. opioid PONV
Koshyari (2019)^20^	45	45	Abdominal hysterectomy	Lidocaine infusion (1.5 mg/kg over 15 min followed by a 2 mg/kg/h infusion until the end of surgery)	Placebo	Postop. opioid Time to first flatus QoR-40
Xu (2017)^19^	60	60	Abdominal hysterectomy	Lidocaine infusion (1.5 mg/kg loading over 10 min before induction of anesthesia, 1.5 mg/kg/h infusion until abdominal wound closure)	Placebo	Pain scores Postop. opioid Time to first flatus
Bryson (2010)^18^	44	46	Abdominal hysterectomy	Lidocaine infusion (bolus infusion 1.5 mg/kg, 3 mg/kg/h continuous infusion)	Placebo	Pain scores Postop. opioid
Yardeni (2009)^13^	30	30	Abdominal hysterectomy	Lidocaine infusion (bolus infusion 2 mg/kg, 1.5 mg/kg/h continuous infusion)	Placebo	Pain scores
Birch (1987)^16^	9	9	Abdominal hysterectomy	Lidocaine infusion (bolus infusion 1.5 mg/kg, 2 mg/kg/h continuous infusion for 2 h)	Placebo	Postop. opioid

PONV, postoperative nausea and vomiting.

In six out of nine RCTs, a lidocaine load (1.5–2 mg/kg) was administered before anesthesia induction, followed by continuous infusion during surgery^13,14,16–18,21^. In RCTs without a lidocaine bolus, lidocaine infusion loading (1.5 mg/kg) commenced 10–15 min before continuous infusion^15,19,20^. Patients in the control group received intravenous infusion of physiological saline in all RCTs. Postoperative analgesia was provided using morphine, fentanyl, or tramadol.

Exclusion criteria included allergy to local anesthetics (five trials)^14,17,19–21^, renal or hepatic dysfunction (five)^14,17–19,21^, severe respiratory disease (four)^14,17,19,21^, BMI >30 kg/m^2^ (four)^14,17,18,21^, BMI >35 kg/m^2^ (two)^15,20^, BMI <18.5 kg/m^2^ (one)^18^, history of sleep apnea (two)^15,20^, cardiovascular disease, hypertension, bradycardia, A-V conduction block, or arrhythmia (eight)^13–17,19,21^, history of preoperative opioid medication, psychiatric conditions, or beta-blockers (eight)^13–17,19,21^, urticaria (two)^17,19^, and ASA grade above II (one)^18^.

### Bias risk analysis

**Figure 2:**
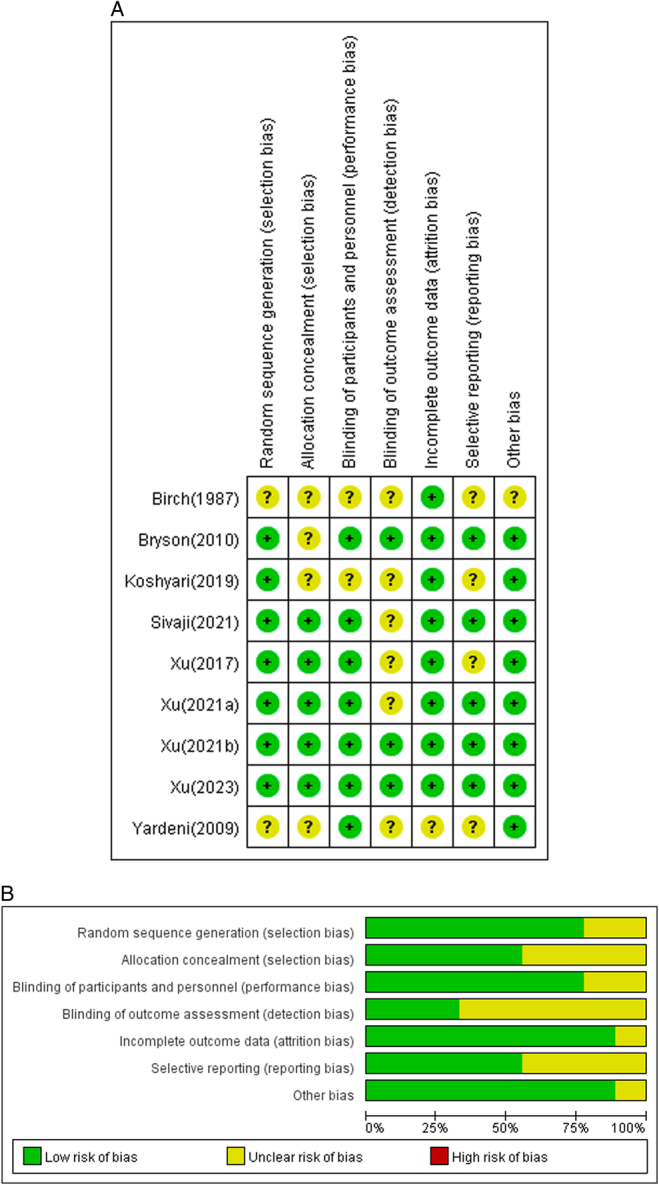
The risk of bias of the included RCTs. (A) Risk of bias graph. Review authors’ judgments about each risk of bias item presented as percentage across all included studies. (B) Risk of bias summary. Review authors’ judgements about each single study, using red, yellow, and green to represent high, medium, and low risk, respectively.

A risk assessment was conducted following the Cochrane Handbook for Systematic Review of Interventions^29^, generating a graph and summary. Figure 2A illustrates the findings regarding the risk of bias in the included RCTs. Among the five RCTs^14,15,17,19,21^, randomization, allocation concealment, and performance bias showed a low risk of bias. Incomplete outcome data had the lowest bias, with only one study categorized as unclear risk^13^, and the remaining studies as low risk. Conversely, blinding of outcome assessment exhibited the highest bias, with only three studies classified as low risk^14,18,21^, and the remaining studies as unclear risk. Additionally, most included studies demonstrated a low risk of bias in terms of attrition bias, detection bias, performance bias, selection bias, and other biases. Approximately 30% of the selected studies exhibited a low risk of bias concerning reporting bias (Fig. 2B). Overall, the included RCTs showed a low risk of bias.

### Primary outcomes

#### Postoperative pain scores at rest and during moving

A total of seven trials evaluated postoperative pain scores at rest. Of these, six trials^13–15,17,19,21^ reported VAS pain scores, and one trial^18^ reported NRS scores. One of the other trials did not provide suitable data for analysis^16^, and one trial^20^ did not report pain scores.

**Figure 3:**
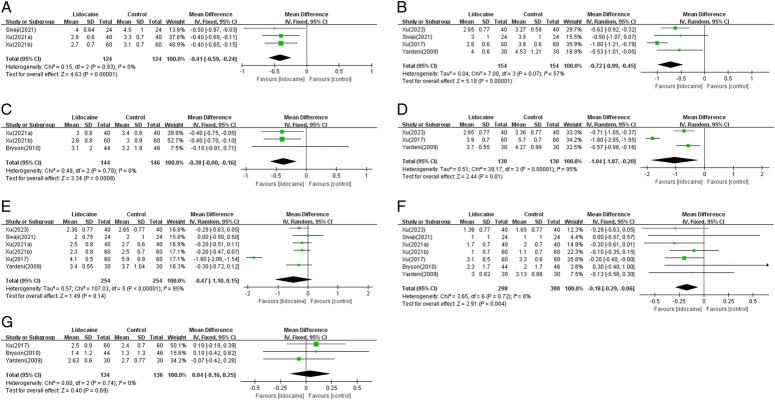
Forest plot comparing lidocaine to control for postoperative pain scores at rest at 2(A), 4(B), 6(C), 8(D), 12(E), 24(F), and 48(G) hours.

Meta-analysis results of these data showed that the postoperative pain scores of the lidocaine group were significantly lower than those of the control group at postoperative 2 h (*n*=3 RCTs, MD=−0.41 cm, 95% CI [−0.59, −0.24], *P*<0.00001) (Fig. 3A), postoperative 4 h (*n*=4 RCTs, MD=−0.72 cm, 95% CI [−0.99, −0.45], *P*<0.00001) (Fig. 3B), postoperative 6 h (*n*=3 RCTs, MD=−0.38 cm, 95% CI [−0.60, −0.16], *P*=0.0008) (Fig. 3C), postoperative 8 h (*n*=3 RCTs, MD=−1.04 cm, 95% CI [−1.87, −0.20], *P*=0.01) (Fig. 3D), and postoperative 24 h (*n*=7 RCTs, MD=−0.18 cm, 95% CI [−0.29, −0.06], *P*=0.004) (Fig. 3F). However, there were no significant differences in pain scores at postoperative 12 h (*n*=6 RCTs, MD=−0.47 cm, 95% CI [−1.10, 0.15], *P*=0.14) (Fig. 3E) and postoperative 48 h (*n*=3 RCTs, MD=0.04 cm, 95% CI [−0.16, 0.25], *P*=0.69) (Fig. 3G). Except for the 4 h postoperative period (*I* ^2^=57%, *P*=0.007), 8 h (*I* ^2^=95%, *P*<0.00001), and 12 h (*I* ^2^=95%, *P*<0.00001), analyses at other time points showed homogeneity. However, due to the limited number of included studies, subgroup analysis for different surgical types at different time points was not performed.

In addition, two studies also reported postoperative pain scores during movement, one of which showed a significant reduction at 4 and 8 h (*P*=0.0001 and *P*=0.048, respectively), but no statistical significance at 12, 24, and 48 h^13^. Another study showed that intravenous lidocaine was ineffective for alleviating postoperative pain during moving at 6, 24, and 48 h^18^.

#### Morphine consumption within 24 h postoperative

**Figure 4:**
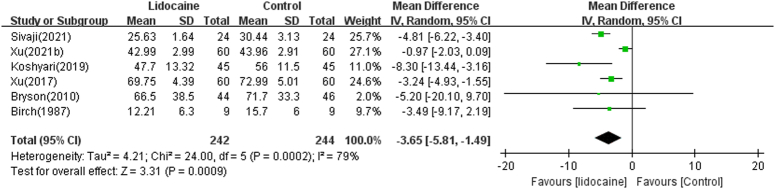
Forest plot comparing lidocaine to control for postoperative morphine consumption within 24 h postoperative.

Six trials reported postoperative morphine consumption within 24 h postsurgery^14–16,18–20^. Compared with the control group, the total postoperative morphine consumption in the lidocaine group was significantly reduced (MD=−3.65 mg, 95% CI [−5.81, −1.49], *P*=0.0009). The pooled analysis showed heterogeneity (*I* ^2^=79%, *P*=0.0002) (Fig. 4). Due to the limited number of included studies, with only one paper each regarding opioid consumption following robotic hysterectomy^15^ and laparoscopic hysterectomy^14^ with lidocaine intravenous injection, subgroup analysis was not conducted. We further conducted a meta-analysis only on morphine consumption after an open abdominal hysterectomy with lidocaine intravenous injection^16,18–20^. Morphine consumption in the lidocaine group was significantly lower than in the control group (*n*=4 RCTs, MD=−4.04 mg, 95% CI [−6.11, −1.97], *P*=0.0001) after open abdominal hysterectomy. Meanwhile, the heterogeneity was significantly reduced (*I* ^2^=12%, *P*=0.33). The surgical types may be one of the sources of high heterogeneity in morphine consumption within 24 h postsurgery.

### Secondary Outcomes

#### PONV

**Figure 5:**
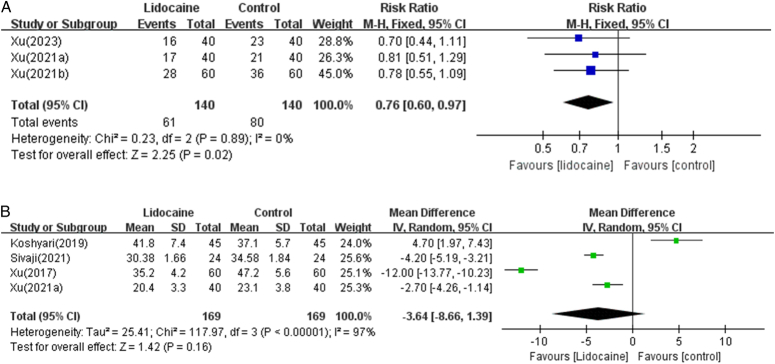
(A) Forest plot demonstrating lidocaine versus control in reducing postoperative vomiting and nausea. (B) Forest plot comparing lidocaine to control for functional gastrointestinal recovery.

Three studies^14,17,21^ provided data on the incidence of PONV. Compared with the control group, the incidence of PONV in the lidocaine group was significantly reduced (*n*=3 RCTs, RR=0.76, 95% CI [0.60, 0.97], *P*=0.02) (Fig. 5A).

#### Postoperative functional gastrointestinal recovery

Functional gastrointestinal recovery was reported in four trials, with one trial^20^ showing no significant improvement, while the other three trials^15,17,19^ demonstrated varying degrees of promotion. Meta-analysis results also showed no statistically significant difference in time to first flatus between the lidocaine group and control group (*n*=4 RCTs, MD=−3.64 h, 95% CI [−8.66, 1.39], *I* ^2^=97%) (Fig. 5B).

#### QoR-40

Only two trials involved the quality of recovery scores (QoR-40), one of which had a median (IQR) quality of recovery scores (QoR-40) of 184 (178–191) in the lidocaine group and 178 (171–180) in the control group (*P*<0.001)^20^, while the other reported QoR-40 scores for each dimension and overall, although there were statistically significant differences in QoR-40 scores between the two groups overall (*P*=0.043), there were no significant differences in dimension scores of QoR-40 for physical comfort, pain, and psychological support^21^. Given the limited number of trials, more trials are needed for statistical analysis.

### Sensitivity analyses and publication bias

The leave-one-out method was used to conduct sensitivity analyses of the primary outcomes, which included postoperative pain scores at rest and morphine consumption. Other primary outcomes all showed stability, except that the pooled results of postoperative pain scores at 12 h changed (from *P*=0.14 to *P*=0.006) after omitting Xu *et al*. (2017)^19^. However, publication bias was not assessed due to the inclusion of fewer than 10 RCTs^30,31^.

## Discussion

### Summary of evidence

This meta-analysis suggested that intravenous injection of lidocaine in patients undergoing hysterectomy under general anesthesia effectively reduced postoperative pain at rest at 2, 6, 8, and 24 h. However, there was no observed benefit in pain relief at rest with intravenous lidocaine injection at 48 h posthysterectomy. Furthermore, the analysis indicated that intravenous lidocaine administration reduced the opioid demand within 24 h after an open abdominal hysterectomy. Additionally, lidocaine injections diminished PONV occurrence in patients undergoing hysterectomy. Finally, there was no observed benefit in the time to first flatus with intravenous lidocaine injection posthysterectomy.

Effective pain management is crucial for reducing postoperative complications and facilitating functional recovery. Although various analgesia methods have been clinically employed, no single mode of analgesia can be deemed optimal to yield satisfactory outcomes^32^. Unlike epidural analgesia, intravenous lidocaine infusion lacks side effects such as motor blockade, urinary retention, and hypotension. When epidural analgesia is impractical or when lidocaine’s invasiveness is unsuitable for procedures like laparoscopic surgery, intravenous lidocaine infusion emerges as an alternative for pain relief. Previous studies have demonstrated the effectiveness of intravenous lidocaine infusion in reducing opioid usage postsurgery^32,33^. Taiym *et al*.^34^ found that lidocaine infusion serves as an effective and safe opioid-sparing analgesic strategy, particularly following gynecologic oncology surgery. Similarly, Alexa *et al*.^33^ reported that intravenous lidocaine infusion accelerates postoperative recovery and reduces the 1-year recurrence rate in terms of mobilization, discharge, and opioid consumption. Herroeder *et al*.^35^ discovered that although intravenous lidocaine infusion reduces postoperative pain in colorectal surgery patients, it does not exhibit a statistically significant difference compared to the control group. In our meta-analysis, we observed a marked decrease in postoperative pain scores at rest at 2, 6, 8, and 24 h following hysterectomy with intravenous lidocaine. Subsequent sensitivity analysis indicated the results are stable. It is worth noting that although there were statistical differences in VAS scores at specific time points between the control group and the intravenous lidocaine group, the differences in average VAS scores between the two groups were relatively small, implicating that the significance of intravenous lidocaine in reducing postoperative in clinical practice pain may not be as ideal as the statistical differences^36,37^. Potential sample size limitations may contribute to this discrepancy. Large-scale studies remain necessary for future research. It should be cautious when interpreting the result of reducing acute postoperative pain following hysterectomy by intravenous lidocaine administration. While some postoperative pain measures did not reach statistical significance at certain time points, this does not imply ineffectiveness of lidocaine. Lidocaine still acts as a sparing agent for opioids, rather than a substitute, reducing morphine equivalent dose while maintaining a similar degree of postoperative pain.

Opioids are commonly administered for pain management following surgery, with Patient Controlled Analgesia (PCA) being a method for patients to self-administer pain relief after hysterectomy^38^. Decreasing opioid dosage is crucial for enhancing postoperative recovery and improving quality of life^39^. However, due to the limited number of published articles, the positive impact of intravenous lidocaine on reducing opioid demand after hysterectomy remains uncertain. Six RCTs involving 486 patients investigated opioid demand outcomes after hysterectomy^14–16,18–20^. The current meta-analysis indicates that intravenous lidocaine administration posthysterectomy is associated with reduced opioid demand within 24 h postsurgery but with high heterogeneity. Sensitivity analysis indicated the results are stable. Subsequent research revealed that postoperative opioid consumption significantly decreased in the lidocaine group following open abdominal hysterectomy within 24 h postsurgery and decreased heterogeneity could be observed^16,18–20^. Furthermore, despite the limited number of studies on the other two surgical types, we should not disregard that intravenous lidocaine administration significantly reduced opioid demand within 24 h after surgery in patients undergoing robotic abdominal hysterectomy^15^ or laparoscopic hysterectomy^14^. High rates of nausea and vomiting pose challenges to effective postoperative recovery. Opioid-related side effects, including PONV, can contribute to delayed recovery. Decreasing opioid usage may lower PONV incidence and enhance postoperative recovery quality. In our meta-analysis, results indicated a notably lower incidence of PONV after hysterectomy in the lidocaine group compared to the control group. Thus, intravenous lidocaine not only reduces opioid consumption but also diminishes PONV occurrence. Consequently, lidocaine can serve as an effective adjunct for postoperative analgesia.

There are multiple reasons for gastrointestinal dysfunction following abdominal surgery, including disruptions in gastrointestinal hormones, administration of opioids and anesthetics, inflammatory responses, and dysfunction of the autonomic nervous system^40^. Intravenous lidocaine injection has been shown to reduce opioid consumption and shorten the duration of intestinal obstruction by preventing inflammatory processes^15,19,41^. However, studies on intestinal motility after perioperative lidocaine infusion present contradictory findings^15,17,19,20^. Sivajia *et al*.^15^ reported that intravenous lidocaine infusion significantly accelerated the recovery of intestinal function compared to the control group, whereas Koshyari *et al*.^20^ discovered that it delayed intestinal function recovery and bloating passage. Xu *et al*.^19^ found that the lidocaine group exhibited a notably shorter time to first bowel sounds and flatus compared to the control group. Additionally, Xu *et al*.^17^ observed in another study that the lidocaine group had a shorter time to first flatus compared to the control group. However, in our meta-analysis, we found that intravenous lidocaine did not significantly influence the time to bowel movement and time to first flatus following hysterectomy. Currently, research on the effects of lidocaine on gastrointestinal recovery is limited.

The meta-analysis has several limitations. Firstly, it is based on nine RCTs, all of which had small sample sizes. Compared to larger trials, small-scale trials are more prone to overestimating treatment effectiveness. The limited number of RCTs makes it challenging to assess the potential impact of publication bias on the results. Additionally, not all outcomes were consistently reported in every trial (see Table 1), and some meta-analyses were based on only three studies, therefore, the conclusions should be interpreted cautiously. Thirdly, heterogeneity in some analyses was high, likely due to different study designs and types of hysterectomy. The results may also be influenced by confounding factors. Lastly, the presence of missing and unpublished data may introduce bias.

## Conclusions

In summary, perioperative intravenous infusion of lidocaine following hysterectomy can reduce the severity of rest pain, postoperative morphine consumption, and the incidence of nausea and vomiting. Intravenous lidocaine injection offers a straightforward alternative to opioid supplementation for postoperative pain relief, which can complement many popular analgesic regimens currently used for hysterectomy. However, considering the low number and overall small sample size of the included studies in the present study, more large-sized RCTs are required to confirm these results. The results of our meta- analysis still should be interpreted cautiously.

## Ethical approval

Not applicable.

## Consent

Not applicable.

## Source of funding

Not applicable.

## Author contribution

F.S.C.: led the conception and design of this study; P.T. and H.Z.L.: was closely involved in data analysis and interpretation and revised the manuscript critically for important intellectual content; Q.X.S. and X.Y.T.: conducted collection and assembly of data and data analysis and interpretation. All authors read and approved the final manuscript.

## Conflicts of interest disclosure

The authors declare that they have no conflicts of interest.

## Research registration unique identifying number (UIN)

The protocol is registered on PROSPERO (CRD42023478834).

## Guarantor

Fengshou Chen and Peng Tang.

## Data availability statement

The data analyzed during the current study are available from the corresponding author upon request.

## Provenance and peer review

Not commissioned, externally peer-reviewed.
